# The First Dose of Fingolimod Affects Circulating Extracellular Vesicles in Multiple Sclerosis Patients

**DOI:** 10.3390/ijms19082448

**Published:** 2018-08-19

**Authors:** Matías Sáenz-Cuesta, Ainhoa Alberro, Maider Muñoz-Culla, Iñaki Osorio-Querejeta, Marta Fernandez-Mercado, Itziar Lopetegui, Mikel Tainta, Álvaro Prada, Tamara Castillo-Triviño, Juan Manuel Falcón-Pérez, Javier Olascoaga, David Otaegui

**Affiliations:** 1 Multiple Sclerosis Unit, Biodonostia Health Research Institute-Donostia University Hospital, 20014 San Sebastian, Spain; xuiny@yahoo.com.ar (M.S.-C.); ainhoa.alberro@biodonostia.org (A.A.); maider.munoz@biodonostia.org (M.M.-C.); inaki.osorio@biodonostia.org (I.O.-Q.); itziar.lopetegilarruskain@osakidetza.eus (I.L.); alvarojose.pradainurrategui@osakidetza.eus (Á.P.); tamara.castillotrivino@osakidetza.eus (T.C.-T.); olascoaga.javier@gmail.com (J.O.); 2 Spanish Network on Multiple Sclerosis, 08028 Barcelona, Spain; 3 Oncology Area, Biodonostia Health Research Institute-Donostia University Hospital, 20014 San Sebastian, Spain; marta.fernandez@biodonostia.org; 4 Department of Neurology, Donostia University Hospital, 20014 San Sebastian, Spain; mikel.taintacuezva@osakidetza.eus; 5 Laboratory of Immunology, Donostia University Hospital, 20014 San Sebastian, Spain; 6 IKERBASQUE, Basque Foundation for Science, 48015 Bilbao, Spain; jfalcon@cicbiogune.es; 7 Exosomes Lab., CIC bioGUNE, CIBERehd, 48980 Derio, Spain

**Keywords:** extracellular vesicles, multiple sclerosis, fingolimod, miRNA, immune regulation, exosomes

## Abstract

Extracellular vesicles (EVs) are membrane-bound particles involved in intercellular communication. They carry proteins, lipids, and nucleotides such as microRNAs (miRNAs) from the secreting cell that can modulate target cells. We and others have previously described the presence of EVs in peripheral blood of multiple sclerosis (MS) patients and postulated them as novel biomarkers. However, their immune function in MS pathogenesis and the effect during the onset of new immunomodulatory therapies on EVs remain elusive. Here, we isolated plasma EVs from fingolimod-treated MS patients in order to assess whether EVs are affected by the first dose of the treatment. We quantified EVs, analyzed their miRNA cargo, and checked their immune regulatory function. Results showed an elevated EV concentration with a dramatic change in their miRNA cargo 5 h after the first dose of fingolimod. Besides, EVs obtained prior to fingolimod treatment showed an increased immune regulatory activity compared to EVs obtained 5 h post-treatment. This work suggests that EVs are implicated in the mechanism of action of immunomodulatory treatments from the initial hours and opens a new avenue to explore a potential use of EVs for early treatment monitoring.

## 1. Introduction

Extracellular vesicles (EVs) are membrane-coated particles of endosomal or plasma membrane origin that are secreted to the extracellular environment [1]. Almost all cell types release EVs and they are found in plasma and other body fluids [2,3]. EVs play an essential role in indirect intercellular communication as their membrane, cytosolic proteins, lipids, and genetic material, including small noncoding RNAs (sncRNA), can be conveyed between cells [4,5]. They are implicated in many biological processes and specifically, EVs have been shown to modulate the immune response by activating or producing inhibitory effects on target cells [6]. 

They have also been described as key players in the pathogenesis of several diseases and it has been postulated that they reflect the inflammatory condition of multiple sclerosis (MS) patients [7]. MS is a demyelinating autoimmune disease of the central nervous system characterized mainly by relapse episodes followed by periods of remission in the first phase of the disease, although there are also patients with primary progressive forms. Specifically, there is an increased EV concentration in the plasma in relapses, which subsequently return to baseline levels during the remission or recovery phase [8,9]. Moreover, the secondary progressive form of the disease, considered to be a low inflammatory stage, has been found to exhibit similar EV counts to those found in healthy controls [10,11]. Furthermore, it has been proposed that the miRNA signature carried by a circulating EV could reflect MS status and so it could be a feasible accessible biomarker for disease monitoring [12].

Currently, the inflammatory condition of MS patients is faced with several immunomodulatory drugs including fingolimod (FGM), the first oral treatment approved for MS. FGM blocks the egress of naïve and central memory T cells from lymph nodes, a crucial step in the inflammation process [13]. Its immediate effect (as soon as a few days after treatment commencement) on the distribution of these lymphocyte subtypes is remarkable and remains stable during treatment [14]. Interestingly, FGM is a specific inhibitor of sphingomyelinase acid, an enzyme that controls EV production [15]. The effect of FGM on myeloid EVs isolated from the cerebrospinal fluid of the animal model of MS was analyzed, reporting a decrease in these EVs [11]. Furthermore, FGM treatment has been shown to restore the number of B-cell- and endothelial-cell-derived EVs, when plasma of MS patients was analyzed [16]. In addition, in a recent publication, a reduction of monocyte-derived EVs in samples obtained from patients after one year of FGM treatment was shown [17]. Nevertheless, to our knowledge, there is no analysis about the effect of FGM on EVs during the first hours after treatment onset.

Accordingly, the aim of this study was to evaluate the immediate effect of FGM, 5 h after the first dose, on peripheral blood circulating EVs of MS patients by analyzing their concentration, sncRNA content, and immunoregulatory function. 

## 2. Results

Data from the studied individuals are shown in Table 1.

### 2.1. EV Characterization

**Figure 1:**
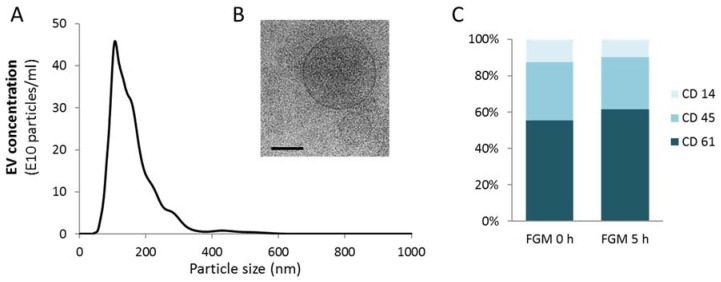
Characterization of circulating EVs in MS patients treated with fingolimod. (**A**) Representative plot showing the size distribution and concentration of an EV sample measured by NTA. (**B**) EV size and morphology by electron microscopy (scale bar = 100 nm). (**C**) Flow cytometry analysis of the distribution of the EV originated from circulating cells (CD61: platelet-derived EVs; CD45: leukocyte-derived EVs; CD14: monocyte-derived EVs) before and 5 h after first dose of FGM. No significant differences were found between the two groups.

Nanoparticle Tracking Analysis (NTA) showed a similar size profile of circulating EVs for both MS patients and controls, with a mode size around 180 nm (Figure 1A). The presence of EVs was confirmed through cryo-electron microscopy (Figure 1B). When analyzing their cell type of origin, most EVs were found to be platelet-derived EVs (CD61+), followed by leukocyte-derived EVs (CD45+) and monocyte-derived EVs (CD14+). This distribution was similar before and 5 h after the first dose of FGM intake (Figure 1C).

### 2.2. FMG Affects the EV Concentration in the Initial Hours

**Figure 2:**
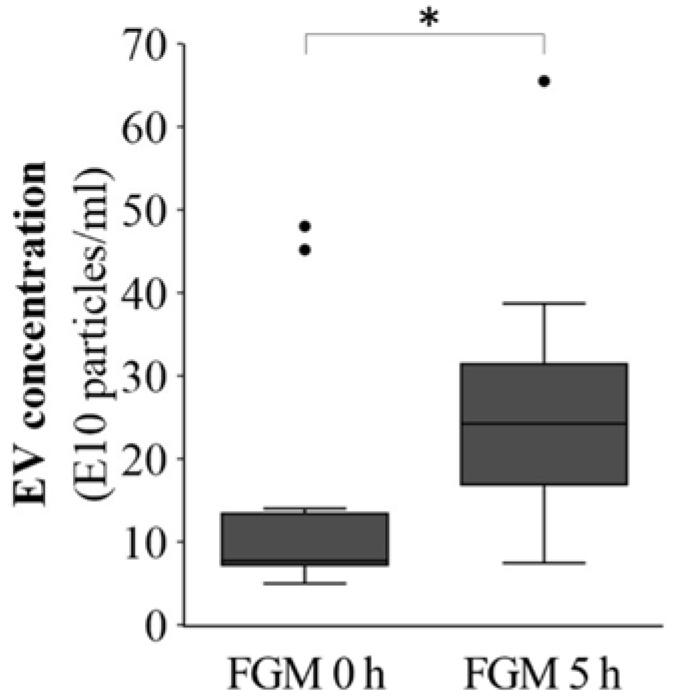
EV concentration before and 5 h after first dose of fingolimod. A significant 1.98-fold change (FC) increase in the concentration of isolated EVs was observed 5 h after the first dose, measured by NTA. * *p* < 0.05. *n* = 11.

The concentration of plasma EVs of MS patients was evaluated by NTA, detecting a twofold increase 5 h after the first dose of FGM (FGM 5 h) when compared to pretreatment concentrations (FGM 0 h) (*p* = 0.045, Figure 2).

### 2.3. FGM Modulates the sncRNA Cargo in the First 5 h

**Figure 3:**
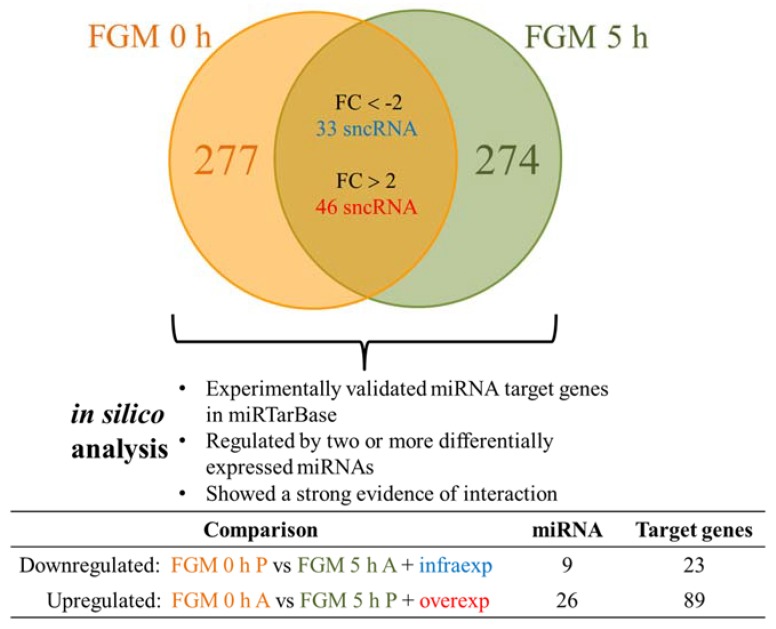
Fingolimod (FGM) modulates the sncRNA cargo in the first 5 h post-treatment. Number of sncRNA present in each condition and those sncRNA infra- or overexpressed at both conditions. Nearly 50% of the sncRNA cargo is different 5 h after the first dose. Besides, among the sncRNAs present in both situations, 46 are overexpressed and 33 underexpressed. In the lower part, the number of miRNAs and their experimentally validated target genes (based on miRTarBase) for the in silico comparisons are shown. A: absent and P: present.

We then decided to focus on the change in EV cargo after the first dose of FGM. The sncRNA content of EVs was analyzed by microarrays. In order to find differences between groups, we selected those sncRNAs present before but absent after the dose intake and vice versa, as well as those sncRNAs that were present at both moments and suffered a modulation in their expression levels (FC > 2 or FC < −2) upon FGM treatment. This analysis showed that 277 sncRNAs were present exclusively at 0 h and 274 only at 5 h (Figure 3). The sncRNA type distribution was similar for both arrays (Table 2). In addition, from those common sncRNAs, 46 were overexpressed (FC > 2) and 33 underexpressed (FC < −2) at 5 h when compared to 0 h (Figure 3).

**Figure 4:**
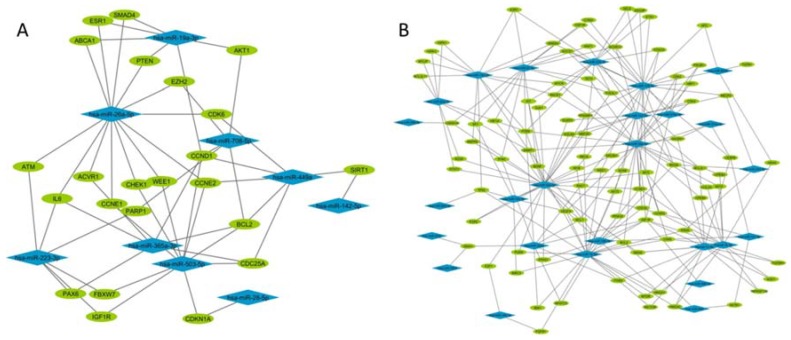
In silico analysis of miRNA cargo of EVs obtained before and 5 h after the first dose of fingolimod. (**A**) Representation of miRNAs (blue diamonds) and target genes (green ovals) interaction network of downregulated and (**B**) upregulated miRNAs 5 h after the first dose of fingolimod.

Next, we studied the potential impact of the modulated sncRNAs by in silico analysis of their predicted target genes in miRTarBase. We found that, among the ones that were downregulated after FGM intake, there were 9 miRNAs with 23 predicted target genes (Figure 3 and Figure 4A). In the opposite sense, among the upregulated ones after the first dose, there were 26 miRNAs with 89 predicted target genes (Figure 3 and Figure 4B). The ontology analysis of both datasets showed that similar biological pathways are potentially affected by the modulation in the EV cargo, being tumor necrosis factor related apoptosis-inducing ligand (TRAIL) signalling, beta 1 integrin family cell surface interactions, epidermal growth factor receptor (EGFR)-dependent endothelin signalling, Arf6 signalling, and S1P1 pathways the most represented ones (Benhamini–Hochberg correction *p* < 0.01). 

### 2.4. 30% of sncRNA Cargo of EVs Is Commonly Expressed in All Conditions

**Figure 5:**
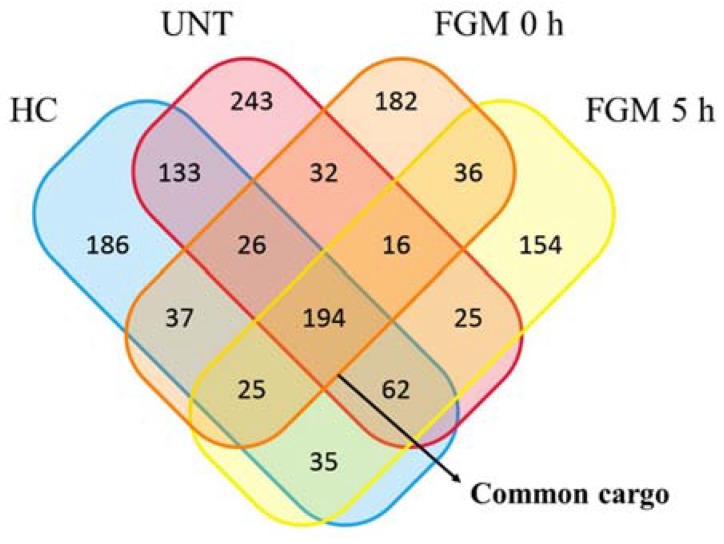
Common cargo of sncRNAs in EVs. Venn diagram shows the number of sncRNAs present in each group, and the common ones among groups. The sncRNAs present in all groups are defined as the “common cargo”. HC: healthy controls; UNT: untreated multiple sclerosis patients; FGM 0 h: fingolimod previous first dose; FGM 5 h: fingolimod 5 h after first dose.

In a second approach, when we compared the EV cargo from FGM 0 h and FGM 5 h patients with EVs from healthy controls (HC) and untreated MS patients (UNT), we identified 194 sncRNAs commonly expressed in the four experimental groups, which were defined as the “common cargo” (Figure 5). It corresponded to 27 to 35% of the total probes of each group.

The ontology analysis of the target genes within the common cargo showed an enrichment of target genes related to focal adhesion. In addition, the sncRNA type distribution was similar in the four groups, most of them being mature miRNAs, followed by immature miRNAs, and finally, different classes of snoRNAs (Table 2).

### 2.5. FGM Affects the Functional Capacity of Circulating EVs

**Figure 6:**
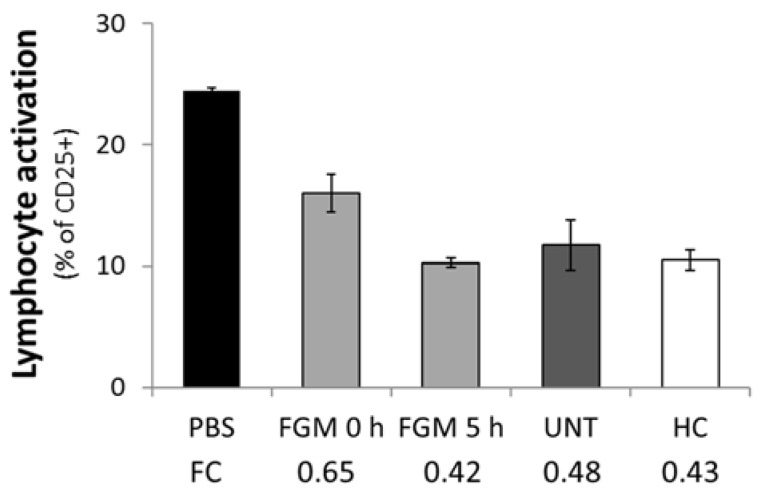
Circulating EVs inhibit lymphocyte activation. Cultured peripheral blood mononuclear cells (PBMCs) were treated with EVs isolated from one donor for each condition (HC, UNT, FGM 0 h, and FGM 5 h) and then activated with PHA addition. In all cases, EVs inhibit lymphocyte activation as shown by flow cytometry analysis of CD25+. Besides, FGM 5 h have impaired inhibition of lymphocyte activation. The fold change (FC) was calculated in respect to the PBS treated sample, and is shown under each bar.

We also set ourselves to determine whether FGM modifies the functional capacity of circulating EVs with respect to lymphocyte activation in the first few hours of treatment. To this end, we cocultured lymphocytes with circulating EVs obtained from FGM 5 h, FGM 0 h, HC, or UNT patients and stimulated their polyclonal activation with phytohaemagglutinin (PHA). Measuring CD25 expression by flow cytometry as a marker of lymphocyte activation, we found that the coculture with EVs from all sources resulted in an inhibitory effect when compared to PBS (baseline activation) (Figure 6). Moreover, the inhibition produced by EVs recovered from 0 h (0.42-fold) was similar to that observed with EVs recovered from UNT and from HC (0.48- and 0.43-fold, respectively), while a lower inhibitory function of EVs from FGM 5 h was reported (0.65-fold). Cell viability and distribution of the lymphocyte populations were not affected upon incubation with EVs. Our data showed that circulating EVs partially inhibit lymphocyte activation without affecting their viability.

## 3. Discussion

This study confirmed the presence of EVs circulating in the blood of MS patients by NTA and microscopy. The presented morphological data are in accordance with preceding findings from our group and others [7,11].

Here, we detected an increase in EV concentration just 5 h after the first dose of FGM, when compared to pretreatment concentrations. According to our previous work and others, EV concentration rises during immunomodulator treatment [10,16]. However, these results were unexpected, since FGM is an analogue of natural sphingosine, which has been demonstrated to play a role in the inhibition of vesicular trafficking [15] and it has been shown that FGM reduces microglia-derived EV concentration in cerebrospinal fluid from mice [11]. Moreover, recently Zinger et al. reported an increased concentration of annexin V+ EVs in untreated MS patients when compared to HC- and FGM-treated patients [16]. Nevertheless, this effect has not been studied in EVs obtained in the initial hours of treatment. In this regard, we hypothesize that there could be a feedback mechanism where initially, the sphingosine analogue effect of FGM could partially inhibit the release of circulating EVs that would result in a later compensatory EV release signal, leading to a subsequent increase in their concentration by 5 h. However, when comparing results between works, the differences in EV sources, isolation methods, and analysis methods should be noted, as these could lead to opposing results.

We also demonstrated that the first dose of FGM changes rapidly and dramatically the sncRNA cargo of EVs. The rapid dynamics of both EV uptake and translation of EV-delivered mRNAs was recently shown [18], supporting that the fast changes that we found after 5 h can have a fast correlation with changes in the recipient cells. On the other hand, changes have been described in the miRNA profile of peripheral blood samples of MS patients after treatment with IFN beta during the first 4 to 12 h [19]. Our results provide a novelty showing that changes produced by FGM are detectable as early as 5 h after the first dose in the sncRNAs carried in EVs. Moreover, we found that about 30% of the cargo was common to all the groups. Through in silico analysis, we observed that the common cargo was involved in focal adhesion, a pathway comprising mechanisms in which cells get attached to basal membranes and migrate through them. This could indicate that there is a fraction of stable sncRNA cargo present in circulating EVs which might be contributing to the attachment and fusion of EVs with target cells.

**Figure 7:**
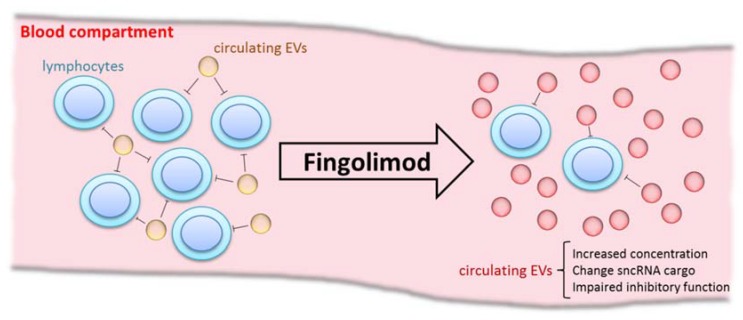
Fingolimod modulates the release and regulatory function of EVs. Before fingolimod (FGM) treatment, lymphocytes circulate freely in the blood stream, maintaining a proinflammatory status that is characteristic of MS. Besides, EV concentration is low even though they have a high immune regulatory function, probably in order to counteract proinflammatory status. After first dose intake of FGM, this scenario changes; lymphocytes are arrested in lymph nodes, conditioning a low inflammatory status and thus the EVs released change to a low regulatory profile according to the circulating microenvironment.

Regarding the function of circulating EVs from MS patients, we demonstrated that they play an inhibitory role in lymphocyte activation. This role has been demonstrated for other EV subtypes, such as those derived from breast milk [20] or cancer cell lines [21] and moreover, Kimura et al. revealed recently the capacity of circulating EVs obtained from MS patients to suppress the induction of Tregs [22]. Importantly, we demonstrated that the lymphocyte inhibitory capacity of circulating EVs is reduced 5 h after the first dose of FGM. Integrating all our results, we could hypothesize that lymphocyte arrest in lymph nodes results in a low inflammatory status, where more EVs with a low regulatory profile are released, according to the circulating microenvironment (Figure 7).

In summary, we have characterized the modulation of the concentration profile, sncRNA cargo, and function of circulating EVs in a group of MS patients during FGM treatment onset. We showed that FGM treatment triggers a rapid initial change in the concentration and sncRNA cargo of EVs which, at the same time, modulates the regulatory effect of those EVs with regard to the activation of lymphocytes. Consequently, this study suggests that EVs play a role in MS and they are implicated in the mechanism of action of the immunomodulatory treatment with FGM since the initial hours, indicating that EVs could be useful biomarkers for early treatment monitoring.

Similar approaches could be applied to study the effect of other immunomodulatory drugs for MS. After the successful results of fingolimod, several sphingosine 1 phosphate receptor (S1PR) modulators have been developed and are now in clinical trials [23]. It would be interesting to test the effect of these drugs on plasma EVs and compare the results. Moreover, in contrast to fingolimod, some of the new compounds are directed to specific S1PR subtypes and therefore, their target cells and effects are different [23]. Taking this into account, we hypothesize that the differences could also affect circulating EVs, but to our knowledge, this possibility has not been addressed yet. Future experiments analyzing EVs in patients treated with S1PR modulators and other drugs will continue to improve our understanding of EVs and their role in MS disease.

## 4. Materials and Methods

### 4.1. Subjects

Eleven MS patients fulfilling the 2010-revised McDonald diagnostic criteria and enrolled to start receiving FGM were recruited at the MS Unit of Donostia University Hospital. All MS patients were in clinical remission. As fingolimod is a second-line treatment, all patients were treated with other drugs before fingolimod. The modification and initiation of the treatment were always controlled by the responsible physicians at the hospital. Patients treated with Natalizumab were at least 3 months without treatment before the fingolimod initiation, while the ones treated with Glatiramer acetate or Interferons did not need this clearance time. 

All patients were subjected to peripheral blood extraction under fasting conditions. The first extraction was performed prior to the first FGM dose (FGM 0 h). Patients receiving the first FGM dose had to stay 6 h at the hospital under blood pressure and cardiac monitoring to check for possible cardiac complications. Thanks to that, an additional blood extraction was performed 5 h postadministration of the first dose (FGM 5 h). The absence of side effects after the completion of the 6-h monitoring was a requisite for including the samples in the study. Samples from 5 UNT MS patients and 8 HC were obtained from the Basque Biobank and processed identically as those from patients. 

After discarding the first milliliter, blood collection was performed by venipuncture with a 21G needle in a 4 ml EDTA tube and a 5 ml citrate tube (BD) and processed within one hour postcollection. All subjects gave written informed consent. The study was approved by the Hospital Ethics Committee (Minutes 1/2014, on 22 January 2014) and methods were carried out in accordance with approved guidelines.

### 4.2. Isolation of Extracellular Vesicles

Blood sample tubes were kept upright and centrifuged at 1250× *g* for 20 min to recover plasma from the supernatant. EVs were isolated as previously described by our group [24]. Briefly, plasma was centrifuged at 13,000× *g* for 2 min and supernatant centrifuged at 20,000× *g* for 20 min to pellet EVs. The 100 µL EV pellet was resuspended with 100 µL of double-filtered (0.22 µm pore filter) DPBS (GIBCO, Thermo Fisher Scientific, Madrid, Spain), to a final volume of 200 µL. Resuspended EVs were stored at −80 °C. Isolated EVs were subjected to cryo-electron microscopy to examine their morphology and size, as previously described [25]. The EVs isolated from EDTA tubes were used for Nanoparticle Tracking Analysis and sncRNA arrays. The EVs coming from Citrate tubes were used for Flow Cytometry measurements and coculture experiments with PBMCs.

### 4.3. Nanoparticle Tracking Analysis (NTA)

NTA was performed to evaluate EV concentration and size distribution with a NanoSight LM10 device (Malvern, Madrid, Spain) equipped with NanoSight NTA software 2.2 (Malvern, Madrid, Spain). Each sample was measured twice and mean EV concentration value calculated. Filtered DPBS was used to test that no background signal was detectable.

### 4.4. Flow Cytometry of Extracellular Vesicles

The labelling and gating of EVs were performed as previously described by our group [24]. Briefly, resuspended EVs were aliquoted and mixed with monoclonal anti-CD61-PE (Cytognos, Salamanca, Spain), anti-CD45-PE (BD Biosciences, Madrid, Spain), or anti-CD14-PE (Cytognos, Salamanca, Spain) and incubated for 20 min at room temperature. Next, labelled EVs were washed with DPBS, centrifuged at 20,000× *g* for 20 min, and acquired in a FACS CantoII flow cytometer (BD, Madrid, Spain). DPBS was used to determinate the background noise and to define the lower detection limit. To define the upper limit of the total EV gate, 1 µm nonlabelled polystyrene latex beads were used (Sigma-Aldrich, Merk, Madrid, Spain). The events that met these criteria were further analyzed for specific labelling (positive for PE marker). We defined CD61+ as platelet-derived EVs, CD45+ as leukocyte-derived EVs, and CD14+ as monocyte-derived EVs. For each sample, total PE positive events were summed and the percentage of EVs of each origin calculated.

### 4.5. Small Noncoding RNA (sncRNAs) Arrays

A 185 µL aliquot of resuspended EVs was used for total RNA extraction using the miRNeasy serum/plasma kit (Qiagen, Madrid, Spain), following manufacture’s protocol. RNA concentrations were measured using a Nanodrop ND-1000 spectrophotometer (Thermo Fisher Scientific, Madrid, Spain). Due to the limited RNA concentration, RNA samples were pooled for microarray analysis, one pool for each group (FGM 0 h, FGM 5 h, UNT, and HC). From each pool, 300 ng of RNA were analyzed by GeneChip miRNA array v4 (Affymetrix, Thermo Fisher Scientific, Madrid, Spain). Raw data normalization was carried out by RMA algorithm in Expression Console software (Affymetrix, Thermo Fisher Scientific, Madrid, Spain). In order to explore the differences in the sncRNAs cargo of EV after the first dose of FGM, the following criteria was applied: (i) those sncRNAs that were present in FGM 0 h but absent in FGM 5 h or infra-expressed (FC > 2) in FGM 5 h and, conversely, (ii) sncRNAs that were absent in FGM 0 h but present in FGM 5 h or overexpressed in FGM 5 h (FC < −2). On the other hand, for the EV common cargo analysis, all the sncRNAs present in each of the 4 groups were selected and compared.

With the aim of assessing the potential regulatory function of differentially expressed probes after FGM intake, we searched for experimentally validated miRNA target genes in the miRTarBase database [26]. The resulting interactions were visualized using Cytoscape, where two additional filtering steps were applied to select only those genes that (i) were regulated by two or more differentially expressed miRNAs, and (ii) showed a strong evidence of interaction according to the database. Finally, the FunRich v2.1.2 software [27] was used to assess the biological processes that were enriched in the network with a Benjamini–Hochberg corrected *p*-value < 0.001. The same procedure was followed for the ontology of the EV common cargo.

### 4.6. Coculture of EVs and PBMCs

Fresh peripheral blood of a healthy donor was collected in sodium heparin tubes and PBMC isolated by phase separation with ficoll. After isolation, 100,000 PBMCs were plated and cocultured with 100 µg of previously isolated plasma EVs in RPMI medium (Thermo Fisher Scientific, Madrid, Spain) in a final volume of 200 µL/well. EVs coming from all conditions (FGM 0 h, FGM 5 h, UNT, HC) were tested, as well as cells cocultured only with PBS as a control. 3 h after PBMCs were activated with (PHA) (ThermoFisher) in a final concentration of 0.1% and incubated for 72 h more. Finally, PBMCs and supernatants were recovered from the wells and processed.

### 4.7. Flow Cytometry of PBMCs

Briefly, PBMCs were washed and pelleted. Each PMBC sample was labelled with an antibody mix (anti-CD16-FITC, anti-CD25-PE, anti-CD14-PE-Cy7, anti-CD19-APC, and anti-CD3-APC-Cy7, all from BD, and 7-aminoactinomycin D from Life Technologies) and incubated for 20 min. Next, cells were washed and acquired in a Guava EasyCyte 8HT flow cytometer (Millipore, Merk, Madrid, Spain). Single staining and isotype controls were used to set the gating strategy. The percentage of each cell population of PBMCs (T, B, NK cells, and monocytes), their activation, and cell viability were analyzed. All conditions were studied in duplicate and the experiment was repeated twice. 

### 4.8. Statistics

ANOVA was applied to assess differences between groups. *p* values < 0.05 were considered significant. The mean and standard deviation were calculated for duplicate measurements. The fold-change was calculated to compare results from different samples.

## Figures and Tables

**Table 1:** Clinical characteristics of individuals studied.

Subject	Age (Years)	EDSS	Evolution Time of MS (Years)	Previous Treatment
UNT1	32	2	10.5	
UNT2	31	0	0.3	
UNT3	37	0	0.0	
UNT4	52	2	13.8	
UNT5	22	0	0.0	
FGM1	36	4.5	6.6	INFβ 1A
FGM2	34	4.5	10.8	INFβ 1B
FGM3	50	6	18.5	INFβ 1A
FGM4	49	4	20.2	NTZ
FGM5	36	3.5	16.8	NTZ
FGM6	40	1	9.2	NTZ
FGM7	50	3.5	22.1	NTZ
FGM8	40	2	12.4	INFβ 1B
FGM9	33	0	9.3	GA
FGM10	30	3.5	4.2	NTZ
FGM11	41	3	20.8	GA
HC1	41			
HC2	28			
HC3	22			
HC4	36			
HC5	26			
HC6	33			
HC7	33			
HC8	42			

EDSS: Expanded Disability Status Scale; MS: Multiple sclerosis; UNT: Untreated MS patient; FGM: MS patient starting with fingolimod therapy; INF: Interferon; NTZ: Natalizumab; GA: Glatiramer acetate; HC: Healthy control.

**Table 2:** sncRNA type distribution for each condition.

sncRNA Type	HC		UNT		FGM 0 h		FGM 5 h		Common Cargo	
Mature miRNA	382	55%	374	53%	262	48%	243	44%	124	64%
Immature miRNA	137	20%	148	21%	115	21%	127	23%	28	14%
snoRNA	140	20%	139	20%	134	24%	134	24%	32	16%
CDBox	29	4%	33	5%	25	5%	30	5%	9	5%
H/ACAbox	6	1%	7	1%	9	2%	7	1%	1	1%
scaRNA	4	1%	4	1%	5	1%	6	1%	0	0%
TOTAL	698	100%	705	100%	550	100%	547	100%	194	100%

HC: Healthy controls; UNT: Untreated MS patients; FGM 0 h: Fingolimod-treated patients before 1^st^ dose; FGM 5 h: Fingolimod-treated patients 5 h after 1st dose.

## Abbreviations

EGRF	Epidermal growth factor receptor
EV	Extracellular vesicle
FC	Fold change
FGM	Fingolimod
HC	Healthy control
MS	Multiple sclerosis
NTA	Nanoparticle Tracking Analysis
PBMC	Peripheral blood mononuclear cell
PHA	Phytohaemagglutinin
sncRNA	Small noncoding RNA
S1PR	Sphingosine 1 phosphate receptor
TRAIL	Tumor necrosis factor related apoptosis-inducing ligand
UNT	Untreated MS patient
